# The Developmental Transcriptome of Bagworm, *Metisa plana* (Lepidoptera: Psychidae) and Insights into Chitin Biosynthesis Genes

**DOI:** 10.3390/genes12010007

**Published:** 2020-12-23

**Authors:** Nur Lina Rahmat, Anis Nadyra Zifruddin, Cik Mohd Rizuan Zainal Abidin, Nor-Azlan Nor Muhammad, Maizom Hassan

**Affiliations:** 1 Institute of Systems Biology, Universiti Kebangsaan Malaysia (UKM), Bangi 43600 UKM, Selangor, Malaysia; p99898@siswa.ukm.edu.my (N.L.R.); anisnadyra.az@gmail.com (A.N.Z.); norazlannm@ukm.edu.my (N.-A.N.M.); 2 Pest Management, FGV R&D Sdn. Bhd., Tun Razak 26400 PPP, Jengka, Pahang, Malaysia; rizuan.z@fgvholdings.com

**Keywords:** *Metisa plana*, transcriptome analysis, high throughput sequencing, expression profiling, metamorphosis, bagworm, chitin

## Abstract

Bagworm, *Metisa plana* (Lepidoptera: Psychidae) is a ubiquitous insect pest in the oil palm plantations. *M. plana* infestation could reduce the oil palm productivity by 40% if it remains untreated over two consecutive years. Despite the urgency to tackle this issue, the genome and transcriptome of *M. plana* have not yet been fully elucidated. Here, we report a comprehensive transcriptome dataset from four different developmental stages of *M. plana*, comprising of egg, third instar larva, pupa and female adult. The de novo transcriptome assembly of the raw data had produced a total of 193,686 transcripts, which were then annotated against UniProt, NCBI non-redundant (NR) database, Gene Ontology, Cluster of Orthologous Group, and Kyoto Encyclopedia of Genes and Genomes databases. From this, 46,534 transcripts were annotated and mapped to 146 known metabolic or signalling KEGG pathways. The paper further identified 41 differentially expressed transcripts encoding seven genes in the chitin biosynthesis pathways, and their expressions across each developmental stage were further analysed. The genetic diversity of *M. plana* was profiled whereby there were 21,516 microsatellite sequences and 379,895 SNPs loci found in the transcriptome of *M. plana*. These datasets add valuable transcriptomic resources for further study of developmental gene expression, transcriptional regulations and functional gene activities involved in the development of *M. plana*. Identification of regulatory genes in the chitin biosynthesis pathway may also help in developing an RNAi-mediated pest control management by targeting certain pathways, and functional studies of the genes in *M. plana*.

## 1. Introduction

**Figure 1:**
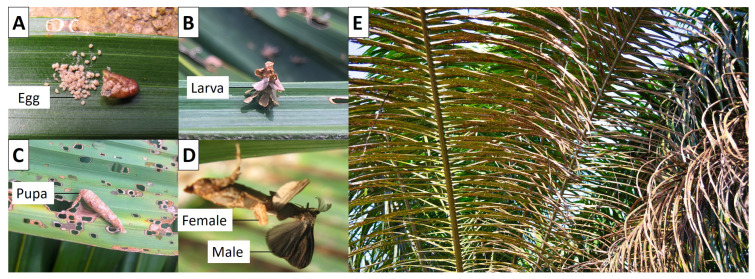
Developmental stages of *M. plana*. (**A**) Cluster of creamy-coloured eggs on the left with its casing on the right. (**B**) Third instar larva. (**C**) Pupa. (**D**) adult on the left and male adult on the right. (**E**) Leaves of oil palm tree upon bagworm outbreak on an oil palm plantation in FELDA Besout 02, Perak, Malaysia (03°46′ N 100°17′ E).

Oil palm is currently Malaysia’s most dominant oil crop with 4,186,914 hectares of oil palm plantations, occupying up to 71.6% of the agricultural land by 2018 [1]. In 2015, the world market value of oil palm reached USD 65.73 billion, and it is predicted to reach USD 92.84 billion in 2021 [2]. *M. plana* (Lepidoptera: Psychidae), bagworm is one of the most destructive insect pest defoliators in the oil palm industry, specifically in Southeast Asia. *M. plana* is a holometabolous insect that completes its development from 80 to 113 days, depending on the climate of the time it grows. *M. plana* development starts from the laying of eggs (Figure 1A) from a mated female, and hatching into neonates, a term used to describe the newly hatched larva. The neonates will feed on oil palm leaves within 1–2 h of emergence to construct a small bag at the posterior end of their body. The case will enlarge progressively with the amount of the plant materials it feeds on and the body size of the larva. Male and female larva will take up to five and seven instar stages, respectively, before metamorphosing into pupa, with the third instar larva being the most active leaf feeder (Figure 1B). The pupa in the casing is mostly found hanging at the back of oil palm leaves (Figure 1C). It has remarkable differences between the male and female’s morphology, and sexual segregation of the pupation site. The male will emerge out of the casing as a fully developed moth while the female stays inside the casing and resembles a maggot with no legs and wings (Figure 1D) [3].

The success of *M. plana* development and metamorphosis depends on several important processes, including the regulation of insect growth hormones (ecdysone and juvenile hormone) and chitin biosynthesis, modification, degradation, and recycling. Chitin forms a significant portion of the integument layer of the exoskeleton and peritrophic matrix of the alimentary canals. Chitin is biosynthesised from the precursor, trehalose, and with continuous enzymatic reaction of eight important enzymes to produce the final polymer chain of UDP-N-acetyl-glucosamine collectively called chitin (Supplementary Figure S1). During moulting, the chitin contents in the cuticle fluctuate with the simultaneous process of chitin biosynthesis and degradation to replace the old cuticle with the new one, especially during larval moult and pupation [4]. Chitin is also required for several important processes during moulting such as in the attachment of cuticle to epidermal cells and the wing disk formation during larva to pupa metamorphosis [5,6]. Chitin metabolic processes were used as insect growth regulators (IGRs) in the development of biorational pesticides as chitin structure is unique to arthropods. Since 1981, targeting chitin for the development of IGRs has been conducted [7], and still largely studied by many researchers in targeting different stages of insect development [8,9,10,11,12].

The outbreak of *M. plana* can cause 50% of leaves defoliation and yield loss of up to 40–47% over two consecutive years of serious infestation (Figure 1E) [13]. The current pest management control of the insect in oil palm plantations involves chemical pesticides, biopesticides, and biological control. Chemical insecticides application through trunk injection technique, ground spraying technique, and aerial spraying includes the use of trichlorfon, cypermethrin, methamidophos, and monocrotophos [14]. The use of biopesticides, such as *Bacillus thuringiensis* (*Bt*) [15,16], endoparasitoid [17], and pheromone mass trapping as a biological control [18] have been reported. Despite these efforts, effective control with economical benefits and safer choice is yet to be found. Efforts towards elucidating the genes involved in the chitin biosynthetic pathway in *M. plana* and analysing its expression trends across multiple stages will be able to help in building safer biorational pesticides targeting the pathway.

In this study, we provided a high throughput transcriptome profiling from four different stages of *M. plana* including egg, third instar larva, pupa, and female adult. A high-quality transcriptome assembly and annotated transcripts were presented to enable a comprehensive comparison between the four stages to further elucidate their differences on the gene level. Differentially expressed transcripts identified among the egg, third instar larva, pupa and female adult and their expression were outlined. Transcripts related to the genes involved in chitin biosynthesis were also identified and their expressions across the developmental stages were investigated. In addition, genetic variation in the transcriptome of *M. plana* was profiled through the identification of microsatellite and single nucleotide polymorphism sites. The transcriptome data will contribute to the knowledge of the molecular components in the developmental aspects of *M. plana*. These data could be further used in the development of safer biorational pesticide or RNA interference-mediated pest management strategies. To our knowledge, this is the first study reporting on the transcriptomics dataset of the different developmental stages of *M. plana*.

## 2. Materials and Methods

### 2.1. Sample Preparation

Egg, third instar larva, pupa and female adult of *M. plana* (Figure 1A–D) were collected from August to November 2018 in an oil palm plantation in FELDA Besout 02, Perak, Malaysia (03°46′ N 100°17′ E). The sampling was consented by the Entomology team of Pest Management, FGV R&D Sdn. Bhd. There was no chemical or biological insecticide applied to the area of sampling two months before the collection. The egg was excised from the casing of the mature female adult using spring scissors (Dumont, Montignez, Switzerland). The third instar larva was identified by the length of its body, which was around 4.0–4.4 mm [3]. The larva, pupa and female adult’ casings that protected them were removed using superfine stainless-steel forceps and spring scissors with straight tips (Dumont). There were two biological replicates for every sample, each with a total weight of 20 mg. The whole-body samples were snap-frozen in the liquid nitrogen and stored at −80 °C freezer for future RNA extraction.

### 2.2. Library Construction and Sequencing

Extraction of total RNA from third instar larva was conducted using RNeasy Mini Kit (Qiagen) by following the recommended protocol. Meanwhile, RNA from egg, pupa and adult female samples were extracted by modifying the procedures suggested by Rio et al. [19] and the protocol supplied with RNeasy Mini Kit (Qiagen, CA, USA). Homogenised tissues from each egg, pupa and adult female samples (20 mg) were dissolved in 1000 µL of Tri Reagent per 100 mg tissue. After adding 200 µL of chloroform, the mixture was vortexed vigorously for 15 s and incubated in room temperature for 3 min. The sample was centrifuged for 10 min at 10,000× *g* and the clear upper phase produced was transferred to a fresh centrifuge tube. A total of 500 µL of isopropanol per each millilitre of the clear phase was added to precipitate the RNA. The mixture was vortexed and incubated for 10 min at room temperature. Then, the mixture was transferred to RNEasy mini spin column (Qiagen) and centrifuged for 15 s at 8000× *g*. The solution was washed two times with 700 µL Buffer RWI and 500 µL Buffer RPE (Qiagen). The purified RNA was collected in RNase free-water. DNA-*free*™ DNA Removal Kit (Invitrogen, CA, USA) was used to remove DNA contamination as instructed in the manufacturer’s protocol. Verification of the RNA yield and quality was assessed using the nucleic acid purity ratio (NanoDrop, Thermo Scientific, FremontCA, USA) while RNA integrity was verified using Bioanalyzer (Agilent Bioanalyzer^®^ 2100, Santa Clara, CA, USA). Following the protocol of the manufacturer, Poly(A) mRNA was isolated using the NEBNext Poly(A) mRNA Magnetic Isolation Module and libraries were prepared using the NEBNext Ultra Directional RNA Library Prep Kit for Illumina (New England Biolabs Inc., Ipswich, MA, USA). The libraries were sequenced using Illumina HiSeq 4000 at 150-bp paired-end (PE) reads by Novogene Med NGS Clinical Laboratory in Tianjin, China.

### 2.3. Transcriptome Assembly

Raw reads obtained were filtered using in-house software (Apical Scientific, Selangor, Malaysia) to remove reads with low quality (Qscore ≤ 5) and reads containing N > 10%. Adapter sequences (Supplementary Table S1) in the raw reads were eliminated using Cutadapt (version 2.3-0, National Bioinformatics Infrastructure Sweden, Uppsala, Sweden) [20]. The quality of the trimmed reads was assessed using FastQC tool (version 04-10-18, Babraham Bioinformatics, Cambridge, UK) [21]. The *de novo* transcriptome assembly was conducted using Trinity RNA-Seq assembly software package (version 2.6.6, Manfred G. Grabherr, MA, USA) withparameter setting: --min_kmer_cov 3 --min_glue 3 --bfly_opts ‘-V 5 --edge-thr=0.1 --stderr’ without a reference genome [22,23]. The transcript abundance was estimated by mapping the trimmed reads back to the assembled transcripts and measured with RNA-Seq by Expectation Maximisation (RSEM) (version 1.2.19, Bo Li & Colin Dewey, Madison, WI, USA) [24] using script align_and_estimate_abundance.pl by Trinity [23]. The transcripts with Fragments Per Kilobase of exon Per Million fragments mapped (FPKM) values below one were filtered from the original transcripts file by using script filter_fasta_by_rsem_values.pl (Part of Trinity) to produce the final transcripts assembly [25]. The quality assessment and completeness of the transcriptome assembly was evaluated using Benchmarking Universal Single-Copy Orthologs (BUSCO) with Lepidoptera core gene set [26].

### 2.4. Transcriptome Annotation and Gene Ontology

**Figure 2:**
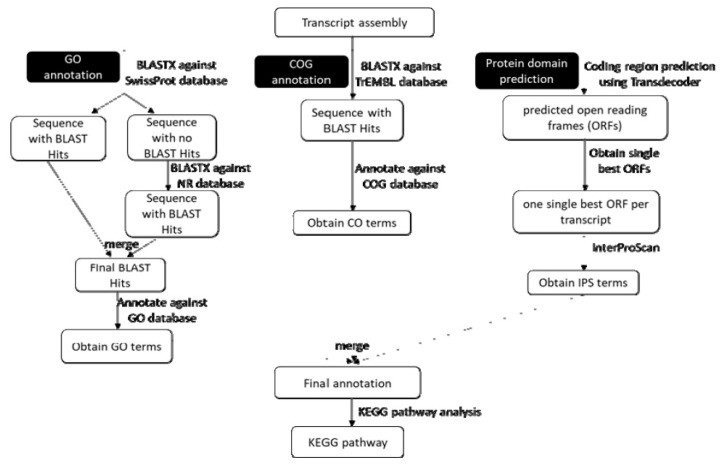
Annotation pipeline. The transcripts were annotated against UniProt, TrEMBL, gene ontology (GO), InterPro and Kyoto Encyclopedia of Genes and Genomes (KEGG) databases.

The annotation was conducted using the pipeline simplified in Figure 2. The assembled transcripts were blasted against UniProt SwissProt database using BLASTX with E-value of 10^−3^ [27,28]. The transcripts that had no blast hits were blasted against NCBI non-redundant (NR) database using DIAMOND BLASTX (E-value of 10^−3^) (version 0.9.24, Benjamin Buchfink, Tübingen, Germany) [29]. The sequences that returned a hit from the search against both databases were merged in Blast2GO (version 5.2.5, BioBam Bioinformatics, Valencia, Spain) [27]. The sequences were annotated with gene ontology (GO) database using Blast2GO with E-value of 10^−3^. The transcripts assembly was also scanned for coding regions using TransDecoder (version 5.5.0, Brian J. Haas, MA, USA) [30]. The minimum length for the translated open reading frames (ORFs) was set to 100 amino acid. Then, the predicted ORFs were filtered to retrieve only one single best ORF per transcripts using the command line --single_best_orf. The predicted ORFs were used to search for protein domains by using InterProScan (version 5.35–74.0, European Bioinformatics Institute, Cambridgeshire, UK) [31]. The transcripts assembly was also blasted to search for sequence similarity against UniProt Translated EMBL (TrEMBL) database [32]. The search was performed using DIAMOND BLASTX (E-value of 1 × 10^−3^). Then, the similarity hits were searched for Eukaryotic Orthologous Groups (KOG) by using the Evolutionary genealogy of genes: Nonsupervised Orthologous Groups (EggNOG) database in Blast2GO software. The Eukaryotic Orthologous Groups (KOG) terms obtained from the EggNOG database and GO terms from InterProScan were merged with the GO terms from GO annotation to obtain the final list of annotated transcripts. The biological pathways in *M. plana* were identified by mapping the annotated sequences to the reference canonical pathways in Kyoto Encyclopedia of Genes and Genomes (KEGG) pathways in Blast2GO [33].

### 2.5. Analysis of Differentially Expressed Transcripts (DETs)

Raw reads counts of each sample were calculated by mapping the raw reads to the transcriptome assembly using Bowtie 2.0 in RSEM software [24]. The abundance estimation was conducted by using the align_and_estimate_abundance.pl script in the Trinity. The gene expression level of each sample was then calculated using edgeR software in R [34]. Pairwise comparison between each developmental stage of *M. plana* was designed as egg against larva, larva against pupa and pupa against female adult. The differentially expressed transcripts (DETs) were selected based on a Log_2_ fold change ≥ 2 and FDR value < 0.001 [35]. GO Gene Set Enrichment Analysis (GSEA) was conducted using Clusterprofiler (version 3.18.0, Guangchuang Yu, Guangzhou, China) to identify overrepresented GO terms with *q*-value < 0.05 [36]. KEGG pathway enrichment analysis was conducted to identify overrepresented metabolic pathways or signal transduction pathways. The *p*-value of each term was generated by using a hypergeometric test to map all the DETs to the terms in the KEGG databases in comparison to the transcriptome background. Enriched KEGG terms were defined as the terms that passed the Bonferroni-corrected *p*-values in the Bonferroni correction [36,37,38,39].

### 2.6. SSR and SNPs Mining

Simple sequence repeats (SSR) in the final transcriptome datasets were identified using the Genome-Wide Microsatellite Analyzing Tool (GMATo) (version 1.2, Xuewen Wang, Zhengzhou, China) (parameter setting: -r 5 -m 2 -x 6 -s 1) [40,41]. Meanwhile, a single nucleotide polymorphisms (SNPs) variant was identified using Picard tools and GATK (version 4.0.3.0, Broad Institute Inc., Cambridge, MA, USA) as suggested in the gatk4-rnaseq-germline-snps-indels workflow from GitHub [42]. High quality reads were aligned against the transcriptome assembly using Bowtie 2.0 [22] with default parameter. The output BAM alignment file was processed using Picard tools AddOrReplaceReadGroups and duplicated reads were removed using GATK tools MarkDuplicates. SplitNCigarReads tool was used to split the reads into exon segments by removing possible Ns reads and hard-clip any sequences overhanging into the intronic regions. The variants were called using HaplotypeCaller and hard-filtered using VariantFiltration in GATK software (FisherStrand (FS) > 30, QualByDepth (QD) < 2) to obtain the final VCF file containing the list of variants [43].

## 3. Results

### 3.1. Sequencing and de novo Assembly

To identify genes that contributed to the development of the morphology and functions of *M. plana* during metamorphosis, the RNA was extracted at four different developmental stages (Figure 1A–D). A total of 243,426,461 reads were obtained with a total size 161.6 GB of 150-bp paired-end (PE) reads through Illumina HiSeq 4000 sequencing platform. There were two steps in verifying the sequencing quality. The in-house software was first used to trim the low-quality reads while Cutadapt software (version 2.3-0) was used to trim the remaining adapter sequences. The final amounts of clean reads were 223,730,116 reads with an average of 24,858,901.78 reads of each stage (Table 1).

The *de novo* assembly of the clean reads was performed using Trinity RNA-Seq assembly software package version 2.6.6. From these results, the initial transcriptome assembly produced by Trinity was filtered using the script filter_fasta_by_rsem_values.pl to filter the transcripts with FPKM values of less than one. The final assembly consisted of 193,668 transcripts and 101,350 predicted ORF. Basic statistics of the assembly library were produced (Table 1). The transcripts length of *M. plana* ranged from 182 to 59,444 bp, with an average length of 906.78 bp. Furthermore, 33.53% of the transcripts were between 300 to 500 bp while 28.40% of the transcripts were between 500 to 1000 bp (Table 1). The *M. plana* assembly was also evaluated using the BUSCO to assess the completeness of the transcripts assembly. The transcriptome assembly showed 97.95% completeness when compared to the Insecta ortholog database.

### 3.2. Annotation and KEGG Classification

**Figure 3:**
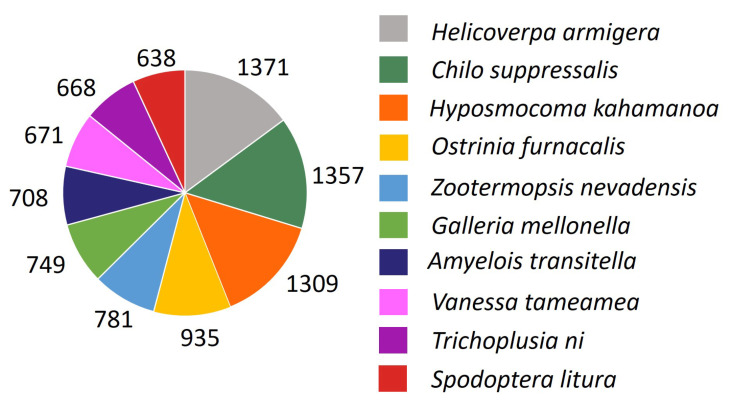
Species distribution of the BLASTX results. Note: This figure shows the top 10 species distribution of transcripts BLASTX results against the SwissProt and NCBI-NR protein database with a cut-off E value 10-3. Different colours represent different species.

The transcriptome assembly was annotated against multiple annotation databases (Table 1). The sequence similarity search of the transcripts against NCBI NR and SwissProt databases using BLASTX returned 59,571 hits. Meanwhile, 56,704 transcripts returned hits when blasted against the TrEMBL database through BLASTX. The top hit species distribution of the matches with known sequences (Figure 3) indicated that the majority of *M. plana* transcripts were homologous with lepidopteran species, including *Helicoverpa armigera* (1371 transcripts), *Chilo suppressalis* (1357 transcripts) and *Hyposmocoma kahamanoa* (1309 transcripts). Other lepidopteran species most represented from the homology search were *Ostrinia furnacalis*, *Plutella xylostella*, *Amyelois transitella* and *Trichoplusia ni*. The alignments were conducted with an E-value threshold of 0.001.

**Figure 4:**
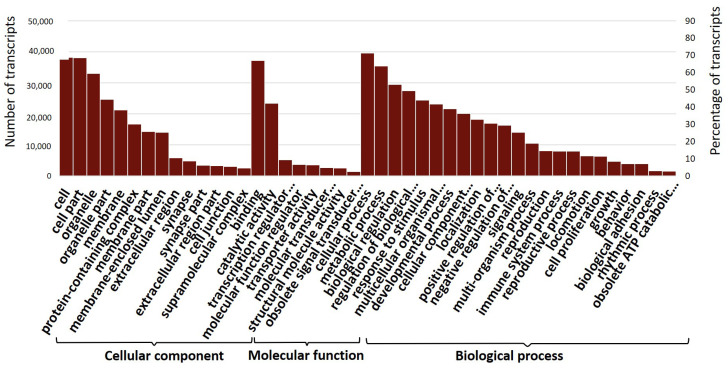
GO classification. Distribution of level 2 GO terms with transcripts percentage more than 1 were visualised using the Web Gene Ontology Annotation Plot (WEGO) web tool. Results are summarised in three main categories: biological process, cellular component, and molecular function. The left axis shows the number of transcripts, while the right axis shows the percentage of transcripts. GO, gene ontology.

Next, Blast2GO was used to assign GO terms and functionally categorise the assembled *M. plana* transcripts, with 46,516 of the assembled transcripts corresponded to at least one GO term (Figure 4). Moreover, 53.16%, 12.88% and 39.25% of the assembled transcripts were classified into biological processes, molecular function and cellular components, respectively. The transcripts involved in the cellular processes (36,911), metabolic processes (31,922), biological regulations (27,270) and responses to a stimulus (22,419) had the highest number of transcripts under the biological process categories. Meanwhile, under the molecular function, binding activity (35,224), catalytic activity (20,778), transcription regulator activity (4635), and molecular function regulator (3194) were among the topmost-abundant sub-groups in the category. Among the subcategories with the highest number of transcripts under the category of cellular components were cell (35,824), cell part (35,785), organelle (30,911), and organelle part (23,414).

**Figure 5:**
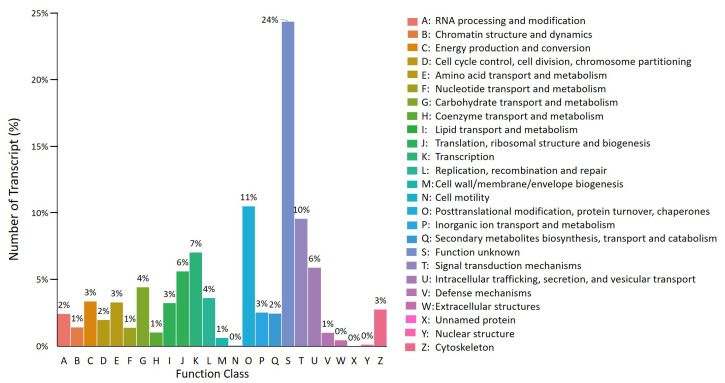
EuKaryotic Orthologous Groups (KOG) functional classification. The final transcriptome assembly was annotated against the KOG database to functionally classify the proteins into 25 functional groups.

The transcriptome assembly was blasted against the Translated EMBL (TrEMBL) protein database, from which the identified proteins were analysed for EuKaryotic Orthologous Groups (KOG) classification. The KOG classification (Figure 5) had classified 50,246 transcripts into 23 functional categories. Transcripts classified under ‘unknown function’ was the highest (16,212, 24%), followed by posttranslational modification, protein turnover, chaperones (7011, 10.55%), signal transduction mechanisms (6384, 9.61%) and transcriptions (4708, 7.08%).

**Figure 6:**
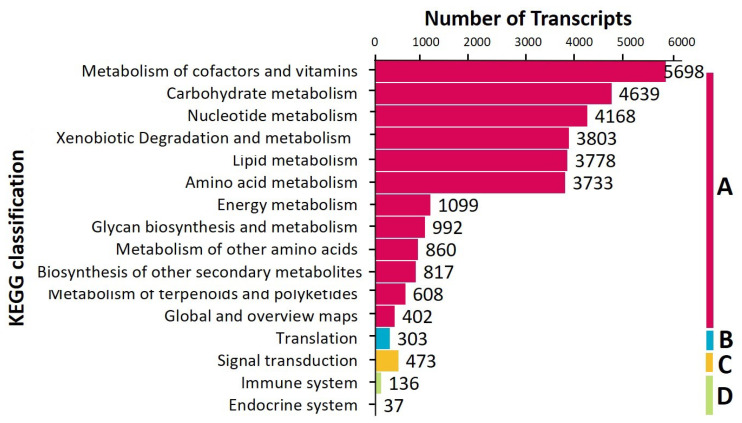
KEGG Pathway classification of *M. plana*’s transcriptomes. (**A**) Metabolism, (**B**) genetic information processing, (**C**) environmental information processing, (**D**) organismal systems.

The annotations were mapped to the KEGG database to produce a total of 1008 known metabolic or signalling KEGG pathways. The pathways were further classified into four KEGG Pathway functional categories (Figure 6): metabolism (30,597), genetic information processing (303), environmental information processing (473), and organismal systems (173). The top three subcategories were metabolism of cofactors and vitamins (5698), carbohydrate metabolism (4639), and nucleotide metabolism (4168).

### 3.3. Analysis of Differentially Expressed Transcripts

**Figure 7:**
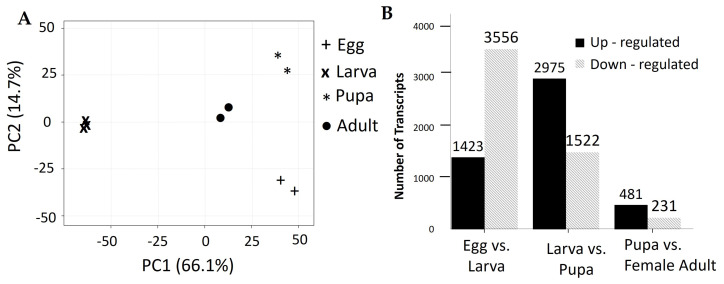
Comparison of transcriptional profile of each developmental stage of *M. plana* (**A**) Principal component analysis (PCA) was conducted using web-based ClustVis software [45] based on normalised (FPKM) and log-transformed count data from nine biological samples of *M. plana*. Principal component 1 (PC1) has 66.1% variance while principal component 2 (PC2) has 14.7% sample variance. (**B**) Number of up-regulated and down-regulated transcripts in each pairwise comparison of egg-larva, larva-pupa and pupa-adult (FDR <0.001, |Log2FC| ≥ 2).

The expression level of all transcripts in the developmental stages of *M. plana* was estimated using Salmon software by mapping the reads back to the final transcriptome assembly [44]. An average of 81.60% of the filtered reads was mapped to the final assembly. The whole transcriptome was subjected to principal component analysis (PCA) using ClustVis software [45] in order to study the compositional bias in the data. The replicates for each developmental stage were clustered together at a high correlation value, indicating the consistency of expression in each replicate at the same developmental stages (Figure 7A).

The pairwise comparison using edgeR software between each stage was conducted: egg against larva, larva against pupa and pupa against adult. Genes with four-fold variation (|Log_2_FC| ≥ 2) in expression with *q*-value of less than 0.001 were considered as differentially expressed transcripts (DETs). A total of 3678 transcripts were differentially expressed in the pairwise comparison of the egg against larva of which 958 transcripts were up-regulated while 2720 transcripts were down-regulated (Figure 7B). Meanwhile, there were 4387 DETs in the pairwise comparison between larva and pupa, with 2431 transcripts being up-regulated while 1956 transcripts being down-regulated. In the pairwise comparison between pupa and female adult, there were 704 DETs, of which 226 transcripts were up-regulated while 478 transcripts were down-regulated.

### 3.4. Expression Profile and Enrichment Analysis

**Figure 8:**
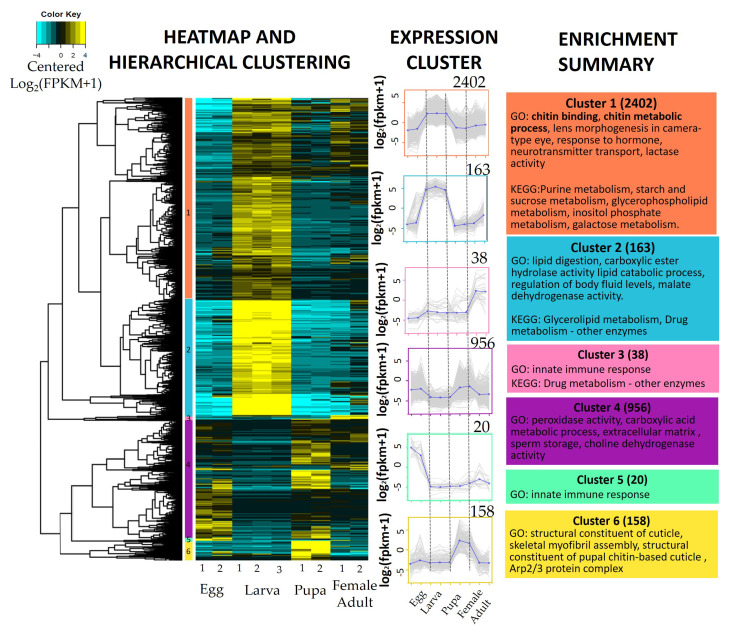
Transcripts expression and cluster enrichment analysis. Heatmap and hierarchical clustering were conducted where 3737 differentially expressed transcripts (DETs) (FDR <0.001, |Log2FC| ≥ 2) were clustered based on Euclidean distance calculated using FPKM-normalised values of each transcript. The hierarchical tree was cut at 0.6 height of the tree to produce six expression clusters with the same expression pattern across the developmental stages. Each cluster was analysed for Gene Ontology Enrichment analysis using Gene Set Enrichment Analysis (GSEA) (*q* < 0.05) and KEGG enrichment analysis using a Hypergeometric test with Bonferroni Correction (Bonferroni-corrected *p* values < 0.05). The enrichment summary lists selected enriched GO terms and KEGG pathways at each cluster (Supplementary Table S2).

Differentially expressed transcripts were clustered in a heatmap using normalised and log-transformed expression value of the DETs (Log_2_FPKM) (Figure 8). Cluster analysis of gene expression was then conducted, assigning these DETs into six clusters (cluster 1 to 6) with same expression patterns across the developmental stages using a hierarchical clustering (--Ptree 0.6). Gene set enrichment analysis (*q*-value < 0.05) and KEGG enrichment analysis (FDR < 0.05) were performed on each cluster (Figure 8) (Supplementary Table S2). Cluster 1 and cluster 2 with 2402 and 163 transcripts, respectively, had high expression in larva but low expression in other developmental stages. These transcripts were associated with enriched GO terms related to chitin structures (chitin-binding, chitin metabolic process, disaccharide transport) and metabolic processes (lipid digestion, lipase activity, galactosylceramidase activity), hormonal responses (response to the hormone, neurotransmitter transport, drug transport) and insecticide resistance (carboxylic ester hydrolase activity, disaccharide metabolic process, ammonium transmembrane transporter activity). The transcripts with high expression in adult were clustered in cluster 3, in which the transcripts were associated with enriched GO terms innate immune response and defence response to fungus and bacterium. In cluster 4, the transcripts were highly expressed in egg and pupa and lowly expressed in larva and adult. The cluster was enriched with GO terms related to oogenesis and embryogenesis, such as peroxidase activity, the carboxylic acid metabolic process and choline dehydrogenase activity. Highly expressed transcripts in the egg stage were grouped in cluster 5, and enriched with innate immune response and antifungal humoral response. The transcripts that had high expression in pupa but low expression in other developmental stages were grouped in cluster 6, with enriched GO terms including a structural constituent of cuticle muscle thin filament and skeletal myofibril assembly.

### 3.5. Identification and Expression Analysis of Transcripts Encoding Chitin Biosynthesis

**Figure 9:**
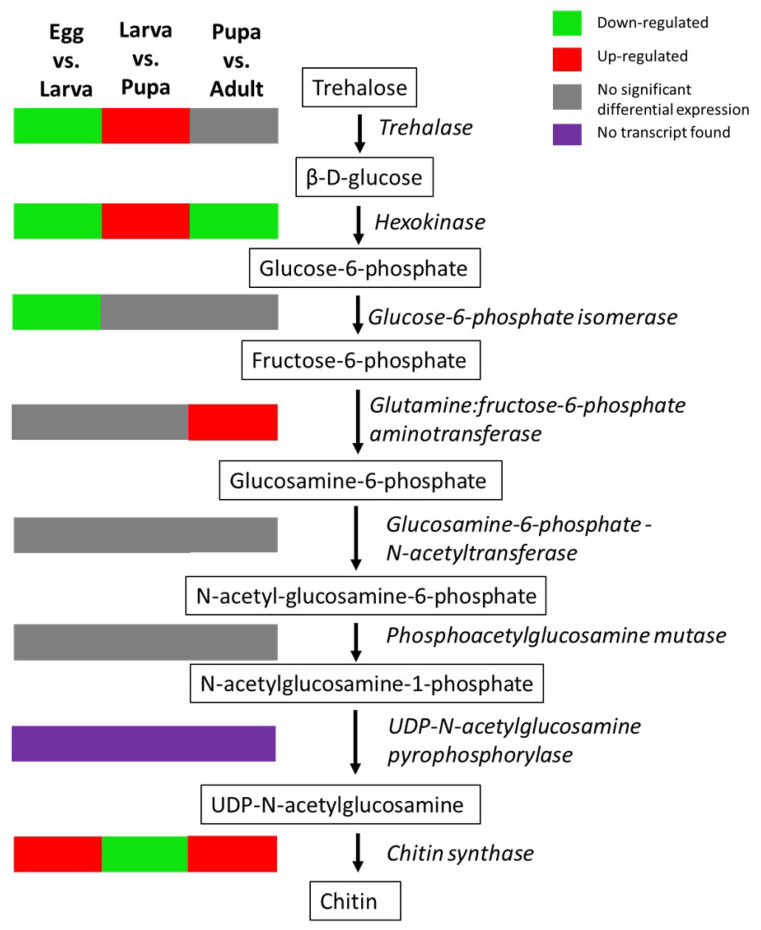
Chitin biosynthesis pathways and expression analysis. The figure shows differentially expressed transcripts (FDR <0.001, |Log_2_FC| ≥ 2) of *M. plana’s* chitin biosynthesis pathway in three pairwise comparisons; egg-larva, larva-pupa and pupa-adult. The pathway starts with trehalose, a sugar compound and ends with chitin polymers. The molecular structure of each substrate was adopted from the previous review on the chitin biosynthesis pathway [46]. The enzymes are in italic. The schematic representation is based on previously established pathways [45,46,47].

Gene mining on the genes involved in the chitin biosynthesis was conducted on the transcriptome of *M. plana*. There were 41 transcripts involved in chitin biosynthesis that were differentially expressed, including chitin synthase, hexokinase, glucose-6-phosphate isomerase, glutamine:fructose-6-phosphate aminotransferase and trehalase (Figure 9; Supplementary Table S3). Trehalase, hexokinase and glucose-6-phosphate isomerase were up-regulated in larva and down-regulated in egg and pupa by more than two-fold. Meanwhile, glutamine:fructose-6-phosphate aminotransferase and chitin synthase were up-regulated in the female adult and down-regulated in the pupa. Apart from that, hexokinase was the only gene in the pathway that was up-regulated in the adult’s transcriptome. The transcripts related to gene glucosamine-6-phosphate N-acetyltransferase and phosphoacetylglucosamine mutase were not significantly differentially expressed while no candidate transcripts with homologs to UDP-N-acetylglucosamine pyrophosphorylase were found in the transcriptome of *M. plana*.

The expression of transcripts related to chitin biosynthesis genes in *M. plana* was compared to other comparative analyses of transcriptome of other insect species at three different comparisons: egg against larva, larva against pupa and pupa against adult (Table 2). Similar expression patterns were seen between *Bombyx mori* and *M. plana*. For example, trehalase genes in both *M. plana* and *B. mori* was down-regulated in egg vs. larva and up-regulated in larva vs. pupa. Meanwhile, *M. plana* had several different expression patterns with *Dendrolimus punctatus*, such as for the comparison between egg and larva, the glucose-6-phosphate isomerase was down-regulated in *M. plana* while the gene was up-regulated in *D. punctatus*. The chitin synthase transcripts in *M. plana* also showed similar expression patterns with *Batocera horsfieldi* and *Hypothenemus hampei* as the expression in all of the species was up-regulated in the comparative differential expression analysis between pupa and adult’s transcriptome.

### 3.6. SSR and SNPs Mining

Genetic diversity of *M. plana* can be observed by the distribution of microsatellite and single nucleotide polymorphisms sequences in its transcriptome. Through SSR identification with GMATo software, 21,516 repeats motifs were found to be repeated at least five times, with the most abundant motifs was di-nucleotide (79.08%), followed by tri-nucleotide (18.39%), tetra-nucleotide (1.97%), petra-nucleotide (0.21%) and hexa-nucleotide (0.15%) (Table 3). Meanwhile, there were 379,895 SNP loci found distributed in all stages of *M. plana*, whereby 159,756 was homozygous SNPs while 220,139 was heterozygous SNP. Egg and pupa stages had the most abundant SNPs loci found (102,392 and 101,570, respectively) while the larva stage had the least SNP sites (79,314). The annotated sequences containing the reported SNR and SNPs locations are presented in Supplementary Table S4.

## 4. Discussion

The severity of *M. plana*’s infestation has led to numerous efforts being done in developing strategic pest control management of *M. plana*. However, a safe, economical, and highly effective insecticide is yet to be found. Currently, the comprehensive genetic information of *M. plana* is limited as studies have focused more on the biology, microstructure, behaviour, and control techniques of the pest. Even though the complete genome sequences of this species are not yet available, the existence of a comprehensive developmental transcriptome of *M. plana* can improve our understanding of its molecular mechanisms and provide a basis for further functional analysis studies. In this study, high-throughput RNA sequencing by Illumina HiSeq 4000 yielded the sequences of *M. plana* transcriptome from the egg, third instar larva, pupa and female adult, producing 223,730,116 reads assembled into 193,686 transcripts. The transcripts were searched against SwissProt, NR, TrEMBL, GO, COG and KEGG databases to produce 46,534 annotated transcripts (Table 1; Figure 3, Figure 4, Figure 5 and Figure 6). Our data were consistent with other developmental transcriptomes of Lepidopteran species, including *Dendrolimus houi*, which obtained 33,720 transcripts and 17,797 annotated transcripts [51,52]. The comparison of the gene expression profile was conducted throughout the developmental stages, which were between egg and third instar larva, larva and pupa, pupa and female adult (Figure 7). A total of 3678 differentially expressed transcripts had been identified, which provided a valuable foundation for future analysis on the key processes in *M. plana* development.

The differentially expressed transcripts were divided into six clusters to demonstrate the gene expression profile across the developmental stages of *M. plana* (Figure 8). The GO enrichment analysis of cluster 5 showed that the transcripts were highly expressed in the egg of *M. plana* but consistently lowly expressed in other stages revolved around innate immune responses. Previous studies have concluded that the egg of lepidopteran insects have a full-range innate immune response as they contain serosa, an extraembryonic epithelium layer. Serosal epithelium secretes cuticle to protect the egg from dehydration, resulting in Toll and IMD pathways upon bacterial infection [53]. Therefore, the egg of *M. plana* might have the innate immune response in the case of pathogen attack; hence, it has higher resilience in a harsher environment. Meanwhile, the transcripts highly expressed in the larva but lowly expressed in egg, pupa and adult in cluster 1 were involved in the key developmental process in larva moulting, such as chitin biosynthesis genes, ecdysone and juvenile hormone-related genes and genes related to digestion. Two insect effector hormones, juvenile hormone and 20-hydroxyecdysone are known to regulate chitin biosynthesis and degradation during the moulting process of larva and pupa. In *B. mori*, chitin deacetylase, an enzyme involved in deacetylation and degradation of chitin, was up-regulated by the juvenile hormone analogue and 20-hydroxyecdysone treatment during the fourth larva moulting period [54] while three chitin synthase genes in *Leptinotarsa decemlineata* were up-regulated by the juvenile hormone during the early stage of each instar [55]. Hence, the moulting of the chitinous structure of the *M. plana* larva might be closely regulated by the two effector hormones as they are being co-expressed together with the chitin metabolic genes in the larva of *M. plana*.

The transcripts clustered together in cluster 6 had a high expression in the pupa but low expression in other developmental stages. These transcripts were associated with cuticle, cytoskeleton and muscle formations, such as actin, cuticular protein and the myosin heavy chain. Several studies have investigated the reformation of muscles and neurons during pupation in the preparation for the insect’s adult stage, such as the reconstruction of the flight muscle and thoracic nervous system. For example, in the *B. mori*, the imaginal disks of the adult appendages develop within the epidermis throughout the larva stages and rapidly construct its wings, legs and antenna during pupation. It can be said that *M. plana* might construct its adult structures and muscles during pupation [56].

In cluster 4, there was a cluster of transcripts having high expression in egg and pupa while low expression in larva and adult. Among them were the transcripts related to glucose dehydrogenase involved in the pentose phosphate pathway, thus indicating the generation of anaerobic energy. The carbohydrate metabolism in eggs undergoing oogenesis and eclosion pupa had been observed in other species such as in silkworm [57].

Chitin is a primary component for building exoskeleton and the peritrophic membrane lining the epithelial midgut of insects [58]. Chitin biosynthesis is crucial for the insect’s moulting and development. Hence, identifying key regulatory genes in the pathway can significantly promote the development of *M. plana’s* pest control and management. A total of 41 differentially expressed transcripts in the transcriptome of *M. plana* were found to have homologs with eight key regulatory enzymes of the chitin biosynthesis pathways (Figure 9; Supplementary Table S3). Many genes in the chitin biosynthesis pathways targeted through RNA interference (RNAi)-mediated knockdown have shown pest control efficiency, with treated insects showing difficulty to moult, failure to break up cuticles, and high mortality rates.

The chitin biosynthesis pathway can be divided into four main phases: (1) hydrolysis of disaccharide precursor, trehalose into simple sugar; (2) acetylation of simple sugar into amino sugar; (3) phosphorylation of amino sugar into a nucleotide sugar; and (4) polymerisation of the nucleotide sugar into chitin polymer (Figure 9) [45,46,47,48,49,50,51,52,53,54,55,56,57]. The enzymes involved in the first and second phase of the pathway, namely trehalase, glutamine-fructose-6-phosphate aminotransferase, and UDP-N-acetylglucosamine pyrophosphorylase are the regulatory enzymes of the pathways. Inhibition of these enzymes through RNAi-mediated knockdown causes chitin loss, moulting inhibition and mortality in insects, including *Tribolium castaneum* (red flour beetle), *Nilaparvata lugens* (brown planthopper), and *Locusta migratoria* (migratory locust) [58,59,60,61,62]. These enzymes were consistently up-regulated in the egg and pupa of *M. plana*, thus indicating the higher chitin biosynthesis genes expression in these two stages. These enzymes might also be involved in wing development as hexokinase was up-regulated in the pupa of *M. plana*, which was similar to the up-regulation of the gene in the pupa of *B. mori*. *B. mori* develops its wings during this stage, thus indicating the presence of de novo synthesis of chitin for the wing development process in both *B. mori* and *M. plana* during the pupa stage [5]. Furthermore, the transcripts related to wing development and formations of the structural constituents of cuticles were exclusively highly expressed in pupa compared to the other stages (Figure 8).

Glucosamine-6-phosphate N-acetyltransferase transcript was not differentially expressed and phosphoacetylglucosamine mutase was not identified in the developmental transcriptome of *M. plana*. However, these two genes were found to be up-regulated in the pupa of *B. mori* and *D. punctatus*. These differences might be due to the failure of achieving full-length transcripts during the de novo assembly of this transcriptome due to presence of many short contigs and the high probability of positive bias in the isoform-level expression analysis [63].

Chitin synthase is a highly conserved enzyme that catalyses the formation of different chitin polymers from UDP-N-acetylglucosamine (UDP-GlcNAc). Chitin synthase-2 (chs-2) was up-regulated in the larva and adult while down-regulated in the egg and pupa of *M. plana*. In *Caenorhabditis elegans*, the chs-2 gene was found to be expressed in the late larva and adult hermaphrodites. In the larva, chs-2 gene is important in chitin deposition at the buccal cavity and grinder of the pharynx of nematodes. The chitinous structure provides rigidity during the pumping of the pharynx, and helps the grinder to breakdown food [64]. Chitin synthase-2 has also been found in the genome of *Eumeta variegata*, a bagworm commonly occurring in Japan [65]. Chitin synthase A gene was found to be highly expressed in the adult’s transcriptome of *Phenacoccus solenopsis* Tinsley (Hemiptera: Pseudococcidae). The gene was suppressed by using RNAi-mediated knockdown of chitin synthase A, resulting in the reduction of the body size and wax coating 72 h after the treatment [66].

A population genetic study is important in the pest management researches, such as the development of genetic markers in the insecticide resistance strains in pests. In *M. plana*, there were 21,516 SSR and 379,895 SNPs found distributed in the transcriptome of the species. SNPs are much more abundant than SSRs as the mutation rate of SNPs (10^−9^ per locus per generation) is much lower than that of SSRs (10^−3^–10^−4^) [67]. Di-nucleotide repeats is the most abundant microsatellite in *M. plana* where AT/TA is the most abundant type of di-nucleotide motifs. This observation is similar to previous reports on *Pieris rapae* [68] and *Orseolia oryzae* [69]. In the SSR analysis of 136 insect genomes, AT/TA were among the most abundance di-nucleotide SSR repeats, alongside with AG/GA/CT/TC and AC/CA/GT/TG [70]. Hence, the distribution of microsatellite in *M. plana* agrees with the natural distribution of microsatellite in other insect species. There was a higher number of heterozygous SNPs than homozygous SNPs in *M. plana*, which is similar in *Dryocosmus kuriphilus* [71]. Currently, there were no ploidy and chromosome study available on *M. plana*. However, it was known that a bagworm species, *Solenobia triquetrella* is an autotetraploid species [72]. Hence, the higher number of heterozygous SNPs loci might be due to the tetraploid characteristic of *M. plana*.

The mining and expression analysis of genes in the chitin biosynthetic pathway will improve the development of biorational pesticide through further functional analysis of the genes in *M. plana*. Functional characterisation of the genes involved in the chitin biosynthetic pathway had been conducted on trehalase [60], chitin synthase [73], glucose-6-phosphate isomerase and UDP-N-acetylglucosamine pyrophosphorylase [74]. In addition, further transcriptomics analysis to discover the presences of parasitoid, bacteria, and viruses in an organism can be done by deep transcriptomics of small RNAs (sRNAs) [75]. Aside from discovering natural enemies of *M. plana* through this technique, it is also possible to discover heritable bacterial symbionts, which help insects in obtaining nutrients [76], resistance against natural enemies [75,76,77,78] or in altering the reproductivity of the host [79]. Hence, further study on the sRNAs to find possible natural enemies or bacterial symbionts of *M. plana* is an interesting approach to enhance the management of the pest.

## 5. Conclusions

In conclusion, a comprehensive and high-quality transcriptome of *M. plana* from the egg, larva, pupa and female adult has been sequenced to provide a quality genetic database of *M. plana*. Transcripts related to insect chitin biosynthesis and degradation were identified and analysed based on the expression pattern to facilitate further development of control strategies, including RNAi-mediated pest control management.

## Figures and Tables

**Table 1:** Data statistics of the raw reads, transcriptome assembly and functional annotation of *M. plana*.

Data Statistics of the Raw Reads		
No. of base pairs before trimming (bp)	67,119,034,800	
No. of base pairs after trimming (bp)	67,068,586,489	
No. of clean reads	223,730,116	
Q30	92.565%	
GC content	44.44%	
**Data statistics of *M. plana*’s transcriptome**		
Type	Initial Assembly	Final Assembly
Total numbers of transcripts	330,017	193,686
Completed BUSCOs	97.95%	97.95%
Total length (bp)	296,149,848	175,630,488
N50 (bp)	1785	1981
Average length (bp)	897.38	906.78
Max length (bp)	59,444	59,444
Min length (bp)	176	182
GC content	38.47%	39.46
**Length distribution of final transcriptome assembly (bp)**		
200	28	
300	64,955	
500	54,967	
1000	28,633	
>2000	21,826	
**Functional annotation statistics**		
Total transcripts	193,686	
SwissProt and NR	59,571	
TrEMBL	56,704	
GO	46,534	
KOG	50,246	
KEGG	1012	

**Table 2:** Comparative differential expression analysis of chitin biosynthesis genes of different insects species as compared to *M. plana* at three comparisons: egg against larva, larva against pupa and pupa against adult.

Chitin Biosynthesis Genes	Comparative Expression Analysis					
	Egg vs. Larva		Larva vs. Pupa		Pupa vs. Adult	
	Down-Regulated	Up-Regulated	Down-Regulated	Up-Regulated	Down-Regulated	Up-Regulated
Trehalase	*M. plana, B. mori* [5]			*M. plana, * *B. mori* [5]		
Hexokinase	*M. plana*		*B. mori, D. punctatus* [5,6,7,8,9,10,11,12,13,14,15,16,17,18,19,20,21,22,23,24,25,26,27,28,29,30,31,32,33,34,35,36,37,38,39,40,41,42,43,44,45,46,47,48]	*M. plana, * *B. mori* [5]	*D. punctatus* [49]	*M. plana*
Glucose-6-phosphate isomerase	*M. plana*	*D. punctatus* [49]	*B. mori* [5]		*D. punctatus* [49]	
Glutamine:fructose-6-phosphate aminotransferase	*D. puctatus* [49]					*M. plana*
Glucosamine-6-phosphate N-acetyltransferase				*B. mori* [5]		
Phosphoacetyl-glucosamine mutase				*B. mori, D. punctatus* [5,6,7,8,9,10,11,12,13,14,15,16,17,18,19,20,21,22,23,24,25,26,27,28,29,30,31,32,33,34,35,36,37,38,39,40,41,42,43,44,45,46,47,48]	*D. punctatus* [49]	
UDP-N-acetyl-glucosamine pyrophosphorylase			*B. mori, D. punctatus* [5,6,7,8,9,10,11,12,13,14,15,16,17,18,19,20,21,22,23,24,25,26,27,28,29,30,31,32,33,34,35,36,37,38,39,40,41,42,43,44,45,46,47,48]	*D. punctatus* [49]	*D. punctatus* [49]	*B. horsfieldi* [50]
Chitin synthase	*D. punctatus* [49]	*M. plana*	*M. plana, D. punctatus* [49]	*B. mori* [5]	*D. punctatus* [49]	*M. plana, B. horsfieldi,H. hampei* [35,36,37,38,39,40,41,42,43,44,45,46,47,48,49]

**Table 3:** Summary of microsatellite and single nucleotide polymorphism distribution in *M. plana*.

Microsatellite (SSR) Sequences Distribution			
SSR Type	Repeat Motif	Number	Frequency (%)
Di-nucleotide	AT/GC/AG/CT Other Types	17,015	79.08
Tri-nucleotide	CGC/TTA/TGA/AAC Other Types	3956	18.39
Tetra-nucleotide	TGTC/GACA/ACAG/GCAC Other Types	424	1.97
Penta-nucleotide	CGAGA/ATTTG/CATTC Other Types	46	0.21
Hexa-nucleotide	CGCGCC/GGGCGC/TTTGAA Other Types	32	0.15
>6 nucleotide	GCGGGCG/GGCGGGC Other Types	43	0.20
Total		21,516	100
	**Statistic of SNP Variants**		
Sample	HomoSNP	HeteroSNP	All
Egg	44,650	57,742	102,392
Larva	31,708	47,606	79,314
Pupa	42,711	58,859	101,570
Adult	40,687	55,932	96,619
Total	159,756	220,139	379,895

## Data Availability

In this study, three types of datasets were generated. The first dataset contains RNA-Seq raw reads of *M. plana*, which were deposited to the NCBI Sequence Read Archive Database (SRR8905606, SRR8905607, SRR8905602, SRR8905604, SRR8905605, SRR8905600, SRR8905601, SRR8905603, SRR8905608) (https://dataview.ncbi.nlm.nih.gov/object/PRJNA531059?reviewer=8k0kgsv7skt1br8co84rq2svt7). The second dataset consists of *M. plana* transcripts, submitted to NCBI Transcriptome Shotgun Assembly Sequence Database (GHOX00000000).

## Supplementary Materials

The following are available online at https://www.mdpi.com/2073-4425/12/1/7/s1, Figure S1: Chitin Biosynthesis Pathway, Table S1: Adapter Sequences used during cDNA library preparation and NGS Illumina HiSeq 4000 sequencing., Table S2: Gene Set Enrichment Analysis based on cluster analysis, Table S3: Transcripts found annotated to eight genes in chitin biosynthesis pathway, Table S4: Annotation of transcripts containing SNPs at each developmental stage.
