# Supplementary material for: The Developmental Transcriptome of Bagworm, Metisa plana (Lepidoptera: Psychidae) and Insights into Chitin Biosynthesis Genes
Source: Genes (Basel). 2020 Dec 23;12(1):7. doi: 10.3390/genes12010007 (PMC7822449; doi:10.3390/genes12010007)
Supplement: Supplementary file 1 [file genes-12-00007-s001.zip › Metisa plana Supplementary Data.pdf]

Supplementary Figure 1: Chitin Biosynthesis Pathway (Adopted fromMerzendorfer, H., Zimoch, L., 2003. Chitin metabolism in insects: Structure, function and regulation of chitin synthases and chitinases. J. Exp. Biol. 206, 4393–4412.)

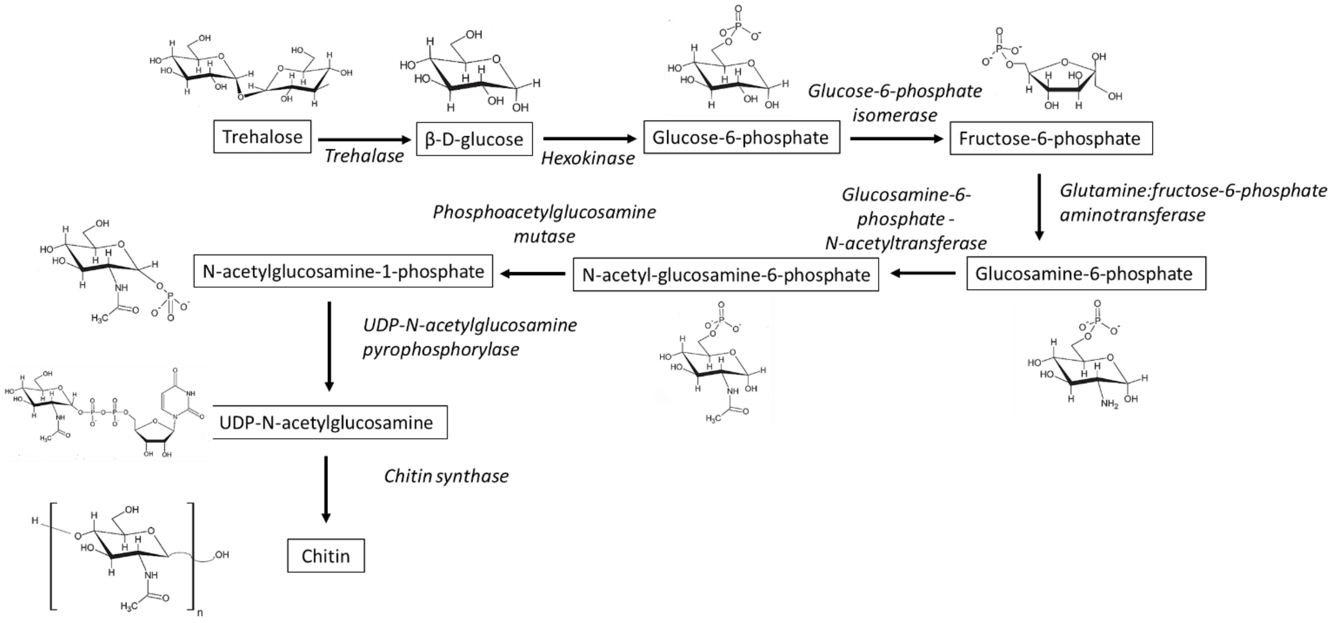

Supplementary Table 1: Adapter Sequences used during cDNA library preparation and NGS Illumina HiSeq 4000 sequencing.

Adapter	Sequence
5' Adapter	5'-AATGATACGGCGACCACCGAGATCTACACTCTTTCCCTACACGACGCTCTTCCGATCT-3'
3' Adapter	5'-GATCGGAAGAGCACACGTCTGAACTCCAGTCACATCACGATCTCGTATGCCGTCTTCTGCTTG-3'

Supplementary Table 2: Gene Set Enrichment Analysis based on cluster.

Cluster 1

GO ID	Description	GeneRatio	p.adjust	qvalue	Transcripts Count
GO:0004565	beta-galactosidase activity	27/1592	2.02E-15	3.25E-16	27
GO:1901805	beta-glucoside catabolic process	23/1592	3.01E-15	3.25E-16	23
GO:0052689	carboxylic ester hydrolase activity	63/1592	3.68E-32	3.18E-32	63
GO:1902494	catalytic complex	24/1592	1.91E-10	8.71E-12	24
GO:0044237	cellular metabolic process	39/1592	2.22E-09	7.40E-11	39
GO:0051692	cellular oligosaccharide catabolic process	23/1592	3.01E-15	3.25E-16	23

GO:0008061	chitin binding	37/1592	7.64E-11	3.89E-12	37
GO:0006030	chitin metabolic process	31/1592	1.82E-11	1.05E-12	31
GO:0015766	disaccharide transport	26/1592	1.95E-11	1.05E-12	26
GO:0015893	drug transport	30/1592	2.55E-13	1.70E-14	30
GO:0004336	galactosylceramidase activity	23/1592	3.01E-15	3.25E-16	23
GO:0006683	galactosylceramide catabolic process	23/1592	4.72E-15	4.54E-16	23
GO:0004348	glucosylceramidase activity	24/1592	8.13E-15	6.39E-16	24
GO:0006680	glucosylceramide catabolic process	24/1592	8.13E-15	6.39E-16	24
GO:0017042	glycosylceramidase activity	23/1592	3.01E-15	3.25E-16	23
GO:0002089	lens morphogenesis in camera-type eye	23/1592	1.30E-11	8.04E-13	23
GO:0016298	lipase activity	22/1592	7.01E-10	2.64E-11	22
GO:0006836	neurotransmitter transport	36/1592	3.18E-17	1.37E-17	36
GO:0016712	oxidoreductase activity, acting on paired donors, with incorporation or reduction of molecular oxygen, reduced flavin or flavoprotein as one donor, and incorporation of one atom of oxygen	32/1592	3.22E-06	5.21E-08	32
GO:1903017	positive regulation of exo-alpha-sialidase activity	23/1592	3.01E-15	3.25E-16	23
GO:0009725	response to hormone	55/1592	2.26E-14	1.63E-15	55
GO:0008663	2',3'-cyclic-nucleotide 2'-phosphodiesterase activity	13/1592	1.22E-09	4.22E-11	13
GO:0030978	alpha-glucan metabolic process	14/1592	5.18E-09	1.55E-10	14
GO:0008519	ammonium transmembrane transporter activity	19/1592	2.63E-09	8.45E-11	19
GO:0044275	cellular carbohydrate catabolic process	18/1592	2.17E-10	9.42E-12	18
GO:0005984	disaccharide metabolic process	14/1592	1.01E-09	3.67E-11	14
GO:0009250	glucan biosynthetic process	14/1592	5.18E-09	1.55E-10	14
GO:0000016	lactase activity	19/1592	6.26E-09	1.81E-10	19
GO:1900408	negative regulation of cellular response to oxidative stress	13/1592	2.86E-10	1.18E-11	13

GO:0015101	organic cation transmembrane transporter activity	18/1592	3.18E-10	1.26E-11	18

Cluster 2

GO ID	Description	GeneRatio	p.adjust	qvalue	Transcripts Count
GO:0044242	cellular lipid catabolic process	10/1985	3.34E-17	4.28E-15	10
GO:0044241	lipid digestion	7/1985	7.73E-13	9.82E-11	7
GO:0052689	carboxylic ester hydrolase activity	11/1985	1.54E-12	1.94E-10	11
GO:0004806	triglyceride lipase activity	6/1985	8.37E-09	1.05E-06	6
GO:0004620	phospholipase activity	6/1985	9.73E-09	1.21E-06	6
GO:0033993	response to lipid	8/1985	1.47E-08	1.81E-06	8
GO:0016298	lipase activity	6/1985	1.49E-08	1.82E-06	6
GO:0016042	lipid catabolic process	7/1985	3.10E-08	3.75E-06	7
GO:0050878	regulation of body fluid levels	5/1985	3.43E-06	0.000411	5
GO:0016615	malate dehydrogenase activity	3/1985	5.58E-06	0.000664	3
GO:0046486	glycerolipid metabolic process	3/1985	7.64E-06	0.000901	3

Cluster 3

GO ID	Description	GeneRatio	p.adjust	qvalue	Transcripts Count
GO:0031640	killing of cells of other organism	8/20	1.31E-18	7.73E-17	8
GO:0045087	innate immune response	8/20	1.61E-12	9.33E-11	8
GO:0050832	defense response to fungus	7/20	4.27E-12	2.43E-10	7
GO:0042742	defense response to bacterium	6/20	2.77E-08	1.55E-06	6

Cluster 4

GO ID	Description	GeneRatio	p.adjust	qvalue	Transcrips Count
GO:0004601	peroxidase activity	12/533	7.13E-10	2.40E-06	12

GO:0019752	carboxylic acid metabolic process	12/533	7.13E-10	2.40E-06	12
GO:0044283	small molecule biosynthetic process	10/533	3.64E-09	1.22E-05	10
GO:0042600	chorion	7/533	7.27E-09	2.44E-05	7
GO:0031012	extracellular matrix	21/533	2.82E-08	9.46E-05	21
GO:0046693	sperm storage	9/533	1.26E-07	0.000422	9
GO:0008812	choline dehydrogenase activity	10/533	2.03E-07	0.000682	10
GO:0019285	glycine betaine biosynthetic process from choline	10/533	2.70E-07	0.000907	10

Cluster 5

GO ID	Description	GeneRatio	p.adjust	qvalue	Transcripts Count
GO:0019732	antifungal humoral response	4/4047	1.13E-11	1.58E-10	4
GO:0031640	killing of cells of other organism	4/4047	2.57E-10	3.34E-09	4
GO:0050830	defense response to Gram-positive bacterium	4/4047	6.86E-08	8.23E-07	4
GO:0045087	innate immune response	4/4047	2.60E-07	2.86E-06	4

Cluster 6

GO ID	Description	GeneRatio	p.adjust	qvalue	Transcripts Count
GO:0042302	structural constituent of cuticle	17/80	5.09E-22	2.03E-19	17
GO:0031012	extracellular matrix	12/80	9.41E-12	3.74E-09	12
GO:0071689	muscle thin filament assembly	6/80	1.65E-10	6.54E-08	6
GO:0014866	skeletal myofibril assembly	5/80	2.01E-10	7.96E-08	5
GO:0008011	structural constituent of pupal chitin-based cuticle	7/80	2.44E-10	9.66E-08	7
GO:0005885	Arp2/3 protein complex	5/80	1.58E-07	6.21E-05	5
GO:0034314	Arp2/3 complex-mediated actin nucleation	5/80	1.81E-07	7.12E-05	5

Supplementary Table 3: Transcripts found annotated to eight genes in chitin biosynthesis pathway.

SeqName	NR_Annotation	Expression Value ($Log_2FoldChange$)		
		Egg vs. Larva	Larva vs. Pupa	Pupa vs. Adult
TRINITY_DN2915_c0_g1_i2	TREA_BOMMOTrehalase OS=Bombyx mori OX=7091 PE=1 SV=1	-3.13602	0.029132	1.129881
TRINITY_DN2915_c0_g1_i4	TREA_BOMMOTrehalase OS=Bombyx mori OX=7091 PE=1 SV=1	-4.87412	4.47895	-1.62793
TRINITY_DN2915_c0_g1_i6	TREA_BOMMOTrehalase OS=Bombyx mori OX=7091 PE=1 SV=1	-12.2877	2.448804	1.30744
TRINITY_DN6124_c0_g1_i10	TREA_APIMETrehalase OS=Apis mellifera OX=7460 PE=1 SV=1	-1.30882	-0.67175	-0.51661
TRINITY_DN6124_c0_g1_i6	TREA_APIMETrehalase OS=Apis mellifera OX=7460 PE=1 SV=1	-2.92124	0.092707	-0.06744
TRINITY_DN6124_c0_g1_i3	TREA_APIMETrehalase OS=Apis mellifera OX=7460 PE=1 SV=1	-1.93432	2.186632	-2.43155
TRINITY_DN6124_c0_g1_i11	TREA_APIMETrehalase OS=Apis mellifera OX=7460 PE=1 SV=1	-4.18027	0.638365	0.262338
TRINITY_DN1357_c0_g1_i1	TREA_BOMMOTrehalase OS=Bombyx mori OX=7091 PE=1 SV=1	2.542527	-2.58847	-0.3325
TRINITY_DN1357_c0_g1_i6	TREA_BOMMOTrehalase OS=Bombyx mori OX=7091 PE=1 SV=1	1.876848	-2.49505	-0.56097
TRINITY_DN1357_c0_g1_i3	TREA_BOMMOTrehalase OS=Bombyx mori OX=7091 PE=1 SV=1	0.438806	-0.17254	-0.81588
TRINITY_DN1357_c0_g1_i8	TREA_BOMMOTrehalase OS=Bombyx mori OX=7091 PE=1 SV=1	-2.56376	0.598989	10.77363
TRINITY_DN1357_c0_g1_i5	TREA_BOMMOTrehalase OS=Bombyx mori OX=7091 PE=1 SV=1	-0.41371	0.9825	#N/A
TRINITY_DN6124_c0_g1_i7	AJK29980.1membrane-bound trehalase	-0.94654	-0.3408	0.277224
TRINITY_DN137622_c0_g1_i1	HXK1_MOUSEHexokinase-1 OS=Mus musculus OX=10090 GN=Hk1 PE=1 SV=3	#N/A	#N/A	#N/A
TRINITY_DN1469_c0_g1_i4	HXK2_DROMEHexokinase type 2 OS=Drosophila melanogaster OX=7227 GN=Hex-t2 PE=2 SV=4	-4.44643	3.145728	0.76468
TRINITY_DN1469_c0_g1_i7	HXK2_DROMEHexokinase type 2 OS=Drosophila melanogaster OX=7227 GN=Hex-t2 PE=2 SV=4	-3.92063	5.938411	-2.8659
TRINITY_DN1469_c0_g1_i3	HXK2_DROMEHexokinase type 2 OS=Drosophila melanogaster OX=7227 GN=Hex-t2 PE=2 SV=4	-4.47667	4.679983	-2.53298

TRINITY_DN2681_c0_g1_i3	HXK2_DROMEHexokinase type 2 OS=Drosophila melanogaster OX=7227 GN=Hex-t2 PE=2 SV=4	-1.0144	0.60795	-0.27291
TRINITY_DN2681_c0_g1_i5	HXK2_DROMEHexokinase type 2 OS=Drosophila melanogaster OX=7227 GN=Hex-t2 PE=2 SV=4	-2.17366	0.408885	0.013199
TRINITY_DN2681_c0_g1_i2	HXK2_DROMEHexokinase type 2 OS=Drosophila melanogaster OX=7227 GN=Hex-t2 PE=2 SV=4	2.595837	#N/A	#N/A
TRINITY_DN2681_c0_g1_i9	HXK2_DROMEHexokinase type 2 OS=Drosophila melanogaster OX=7227 GN=Hex-t2 PE=2 SV=4	-2.17961	#N/A	#N/A
TRINITY_DN2681_c0_g1_i7	HXK2_DROMEHexokinase type 2 OS=Drosophila melanogaster OX=7227 GN=Hex-t2 PE=2 SV=4	-2.22398	0.607288	1.697639
TRINITY_DN775_c0_g1_i4	G6PI_DROSIGlucose-6-phosphate isomerase OS=Drosophila simulans OX=7240 GN=Pgi PE=3 SV=1	-1.31079	1.676421	0.799702
TRINITY_DN775_c0_g1_i7	G6PI_DROSIGlucose-6-phosphate isomerase OS=Drosophila simulans OX=7240 GN=Pgi PE=3 SV=1	-2.82185	1.09908	0.718165
TRINITY_DN7256_c0_g1_i3	GFPT1_RATGlutamine--fructose-6-phosphate aminotransferase	#N/A	#N/A	-10.915
TRINITY_DN7256_c0_g1_i7	GFPT1_RATGlutamine--fructose-6-phosphate aminotransferase	-1.53844	-0.21209	1.314311
TRINITY_DN7256_c0_g1_i4	GFPT1_RATGlutamine--fructose-6-phosphate aminotransferase	-1.46081	-0.30843	3.200951
TRINITY_DN3901_c0_g1_i1	GNA1_DROMEProbable glucosamine 6-phosphate N-acetyltransferase OS=Drosophila melanogaster OX=7227 GN=Gnpnat PE=2 SV=1	-0.88195	0.06111	0.794894
TRINITY_DN2971_c1_g1_i8	AGM1_PIGPhosphoacetylglucosamine mutase OS=Sus scrofa OX=9823 GN=PGM3 PE=1 SV=2	-0.74746	1.233152	0.709444
TRINITY_DN2971_c1_g1_i4	AGM1_PIGPhosphoacetylglucosamine mutase OS=Sus scrofa OX=9823 GN=PGM3 PE=1 SV=2	-9.5357	-0.79988	-0.19831
TRINITY_DN2971_c1_g1_i6	AGM1_PIGPhosphoacetylglucosamine mutase OS=Sus scrofa OX=9823 GN=PGM3 PE=1 SV=2	-0.06303	-1.20814	0.815162
TRINITY_DN1351_c0_g1_i2	CHS2_CAEELChitin synthase chs-2 OS=Caenorhabditis elegans OX=6239 GN=chs-2 PE=1 SV=1	1.814428	-3.85722	3.373191
TRINITY_DN1351_c0_g1_i5	CHS2_CAEELChitin synthase chs-2 OS=Caenorhabditis elegans OX=6239 GN=chs-2 PE=1 SV=1	-0.31948	-1.33343	3.681954
TRINITY_DN1351_c0_g1_i9	CHS2_CAEELChitin synthase chs-2 OS=Caenorhabditis elegans OX=6239 GN=chs-2 PE=1 SV=1	#N/A	#N/A	#N/A

TRINITY_DN1351_c0_g1_i11	CHS2_CAEELChitin synthase chs-2 OS=Caenorhabditis elegans OX=6239 GN=chs-2 PE=1 SV=1	3.400059	-3.38524	4.676408
TRINITY_DN1351_c0_g1_i3	CHS2_CAEELChitin synthase chs-2 OS=Caenorhabditis elegans OX=6239 GN=chs-2 PE=1 SV=1	-0.90486	-2.55861	4.382957
TRINITY_DN1351_c0_g1_i14	CHS2_CAEELChitin synthase chs-2 OS=Caenorhabditis elegans OX=6239 GN=chs-2 PE=1 SV=1	4.821613	-4.15501	3.11665
TRINITY_DN1351_c0_g1_i15	CHS2_CAEELChitin synthase chs-2 OS=Caenorhabditis elegans OX=6239 GN=chs-2 PE=1 SV=1	0.396969	-0.87351	5.535983
TRINITY_DN1351_c0_g1_i13	CHS2_CAEELChitin synthase chs-2 OS=Caenorhabditis elegans OX=6239 GN=chs-2 PE=1 SV=1	2.053691	-0.95658	3.796243
TRINITY_DN1351_c0_g1_i10	CHS2_CAEELChitin synthase chs-2 OS=Caenorhabditis elegans OX=6239 GN=chs-2 PE=1 SV=1	-1.93208	-1.57574	3.022684
TRINITY_DN5630_c0_g1_i3	CHS2_CAEELChitin synthase chs-2 OS=Caenorhabditis elegans OX=6239 GN=chs-2 PE=1 SV=1	-7.01104	7.230801	-3.45538
